# Design, Optimization, and Characterization of Freeze-Dried Emulsions Based on Sodium Alginate and Whey Protein Isolate Intended for Cosmetic and Dermatological Applications

**DOI:** 10.1021/acsomega.5c02358

**Published:** 2025-06-03

**Authors:** Weronika Walendziak, Timothy E. L. Douglas, Justyna Kozlowska

**Affiliations:** † Faculty of Chemistry, 49577 Nicolaus Copernicus University in Torun, Gagarina 7, 87-100 Torun, Poland; ‡ School of Engineering, 4396 Lancaster University, Gillow Avenue, Lancaster LA1 4YW, United Kingdom

## Abstract

Traditional water-based emulsions dominate personal care products, offering minimal skincare benefits but consuming significant amounts of water resources. This sustainable prototype of biopolymer-based skincare products reduces water usage due to the potential to reuse water sublimed during freeze-drying and enhances biopolymer-based materials’ performance. This study presents the development and characterization of freeze-dried emulsions formulated with biopolymers, specifically sodium alginate and whey protein isolate, aimed at cosmetic and dermatological applications. Emulsions were modified with cryoprotectants, including glycerin, propylene glycol, sorbitol, mannitol, and trehalose, as well as oils (sunflower oil or sea buckthorn oil), beeswax, and Span 80 as an emulsifier. The methodology involved varying the time and speed of emulsion homogenization to optimize the size distribution of the oily phase droplets. The prepared freeze-dried emulsions were characterized by scanning electron microscopy (SEM), mechanical properties, residual moisture content, porosity, and density measurements. The physicochemical properties of obtained matrices significantly depended on the concentration of WPI, aqueous-to-oily phase mixing ratios, and the addition of different types and concentrations of cryoprotectants, oils, and beeswax. The results revealed that the obtained materials exhibited promising porosity (59–95%) and density (varying from 116 to 308 mg/mL), low residual moisture content (from 2.3 to 10.9%), and favorable mechanical properties (ranging from 240 kPa to 1.7 MPa), positioning them as novel materials with potential for skin application. Optimization and a combination of existing technologies for a sustainable, functional skincare solution show a superior performance over conventional formulations in terms of shelf life, microbial stability, reduced preservatives, and more efficient transport and storage.

## Graphical abstract

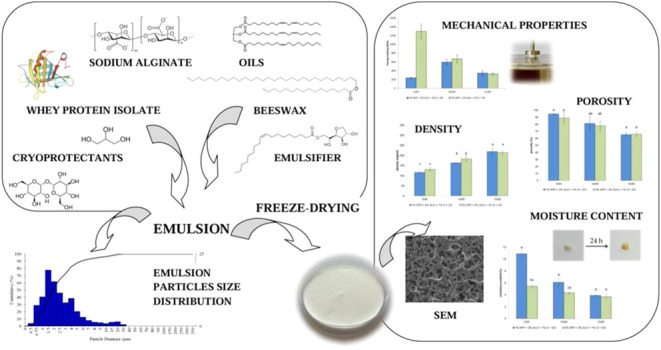

## 1 Introduction

Emulsions are dispersed systems composed of two mutually immiscible liquids: the internal (the dispersed phase) is finely dispersed in the external (the continuous phase) with the help of surface-active agents. There are several distinguished types of emulsions.^1,2^ The oil phase dispersed in the aqueous phase constitutes an oil-in-water emulsion (O/W), whereas a water-in-oil (W/O) system is when the oil is in the continuous phase and the water is in the dispersed phase. Multiple emulsions are also possible, including water-in-oil-in-water (W/O/W) and oil-in-water-in-oil (O/W/O) emulsions, whose smaller droplets are dispersed in larger ones.^3^ The basis of the oily phase may be various oils, such as sunflower oil and sea buckthorn oil, owing to their beneficial properties as skin conditioning agents. Sunflower oil, rich in vitamin E, oleic acid, and linoleic acid, provides skin hydration, barrier repair, and antioxidant protection, making it a widely used emollient in skincare formulations.^4^ Sea buckthorn oil, known for its high content of carotenoids, flavonoids, and essential fatty acids, offers strong anti-inflammatory, regenerative, and antioxidant effects, promoting skin conditioning and protection against oxidative stress.^5^ Beeswax contributes to the emulsion due to its stabilizing properties.^6^ Essential components of emulsions are emulsifiers that lower the surface tension between two phases and stabilize the system by ensuring its thermodynamic stability. These compounds are made of the hydrophilic part (“head”) and the hydrophobic part (“tail”), which are appropriately placed around the internal phase droplets. However, surfactant molecules tend to quickly adsorb and desorb from the droplets, affecting the emulsions’ stability. Moreover, the phases of emulsions can be separated through flocculation, coagulation, coalescence, creaming, sedimentation, and Oswald ripening. The average droplet size and distribution play an important role in these processes along with the emulsions’ pH, viscosity, and added ingredients.^3,7^ The thermodynamic instability may be overcome by freeze-drying of emulsions.^8^ During freeze-drying under low pressure and reduced temperature, water is removed via sublimation from frozen samples.^9^ Resulting porous materials combine the advantages of both forms: emulsions and freeze-dried matrices. However, some mechanical stress may occur during the freeze-drying process, potentially destabilizing the emulsified system. Therefore, several parameters must be carefully and thoroughly chosen, such as the proper selection of emulsifiers and cryoprotectants. Cryoprotectants prevent damage from irreversible aggregation and fusion of internal phase droplets during freezing. Many polyols and sugars (including monosaccharides, disaccharides, and polysaccharides), such as glycerin, propylene glycol, sorbitol, mannitol, and trehalose, play the role of cryoprotectants. Emulsions have found applications in cosmetic,^10,11^ pharmaceutical,^12,13^ medical,^14,15^ and food industries.^16,17^ However, there are not many studies regarding freeze-dried emulsions, including their applications in medical (for vessel clips^18^) and pharmaceutical (as adsorbents for pharmaceutical pollutants,^19^ for intravenous injection of bufadienolides,^20^ and vaccine adjuvants^21^) industries.

Whey protein isolate (WPI) and sodium alginate (ALG) are widely used biopolymers in various applications due to their complementary functional properties and biocompatibility. Whey protein isolate, a highly purified form of whey protein with a protein content exceeding 90% and richness in essential amino acids, is derived from milk whey through filtration. It exhibits remarkable emulsifying, stabilizing, and film-forming properties, making it highly suitable for creating structured emulsions.^22^ WPI contributes to forming stable interfacial layers around dispersed oil droplets, enhancing the physical stability of emulsions.^23^ Its proteinaceous nature also aids in water binding and gelation, critical for maintaining the freeze-dried systems’ structural integrity.^24^ However, sodium alginate, a naturally occurring polysaccharide extracted from brown seaweed, is composed of repeating units of β-d-mannuronic acid and α-l-guluronic acid. It is a thickening, gelling, and stabilizing agent in various formulations.^25^ In emulsion-based systems, sodium alginate contributes to the viscosity and stability of the aqueous phase, providing a matrix-enhancing encapsulation of oil droplets and reducing water mobility.^26,27^ This characteristic is especially advantageous in freeze-dried formulations, where moisture control is essential for maintaining the product stability. Combining WPI and sodium alginate in emulsion-based systems offers synergistic advantages for developing freeze-dried products with tailored textural properties. The structural network formed by these biopolymers plays a crucial role in water immobilization, cryoprotection, and sublimation behavior during the freeze-drying process.

A robust ecological trend can now be seen in cosmetic chemistry (the so-called ecological cosmetics) in terms of both formulation^28,29^ and packaging.^30,31^ Research focuses on biodegradable packaging and natural ingredients; however, no one seems to notice the escalating global water crisis.^32^ Water is the main constituent of numerous cosmetic and personal care products including emulsions. It can be found in skin, body, hair, oral, and sun care products. However, water has no cosmetic effect on the skin; it is only the base component and solvent of other ingredients in most cosmetic forms. Due to the rapidly shrinking resources of clean and accessible water, reducing water usage for formulating products is a responsible attitude toward climate change and the global water crisis.

Developing a methodology for obtaining a prototype with reduced water consumption is one possible solution to reduce the overuse of water in cosmetic chemistry. These advanced materials also have many other advantages, including an increased shelf life and stability, reduced proneness to microbial growth (potentially reducing preservatives’ content), and more accessible storage and transport (due to the reduced weight). Moreover, dry-form materials are practical and travel-friendly; they are lighter and may be packed into hand luggage when passing through airport security. This enhanced cosmetic form is eco-friendly not only because of sustainable water management but also because of the packaging; materials in the dry form allow the reduction of plastic packaging and, thus, plastic waste. Reducing the amount of plastic packaging will be possible because the prototype is stored in a dry form and has a smaller volume and size than traditional emulsions. Moreover, this optimized form presents opportunities in product development that require reduced water usage to prepare and apply the material.

Therefore, the main goal of this research was to develop a preparation methodology for freeze-dried emulsions based on biopolymers (sodium alginate and whey protein isolate), cryoprotectants (glycerin, propylene glycol, sorbitol, mannitol, and trehalose), oils (sunflower oil and sea buckthorn oil), beeswax, and emulsifiers (Span 80). Different times and speeds of emulsion homogenization were investigated in order to determine the narrow size distribution of the smallest dispersed phase droplets in the emulsion continuous phase. Freeze-dried emulsions were characterized via scanning electron microscopy (SEM), mechanical properties, residual moisture content, porosity, and density measurements. Prepared freeze-dried emulsions are advanced, highly effective materials with potential skin applications intended for cosmetic and dermatological applications.

## 2 Materials and Methods

### 2.1 Materials

Sodium alginate was acquired from BÜCHI Labortechnik AG (Flawil, Switzerland). Span 80, d-sorbitol, and beeswax were obtained from Sigma-Aldrich (Poznan, Poland). Glycerin and propylene glycol were supplied from Chempur (Piekary Slaskie, Poland). d-Mannitol and d-(+)-trehalose dihydrate were received from Pol-Aura (Poznan, Poland). Isopropanol was purchased from Stanlab (Lublin, Poland). All of the mentioned chemicals were of analytical grade. Sunflower oil and sea buckthorn oil were obtained from Nanga (Zlotow, Poland). Whey protein isolate (BiPRO) with 97.7% protein and 75% β-lactoglobulin in DM was obtained from Davisco Foods International Inc. (Eden Prairie, MN).

### 2.2 Preparation Method of Materials

Materials based on whey protein isolate, sodium alginate, cryoprotectants, and oily substances were prepared by freeze-drying of the O/W emulsions. Compositions of fabricated materials are presented in Table 1. Emulsions were obtained with three oily-to-aqueous mixing ratios (5/95, 10/90, and 15/85). Different concentrations of WPI (1 or 3%), different types and concentrations of cryoprotectants (glycerin, propylene glycol, sorbitol, mannitol, and trehalose) (1 or 3%), different types of oils (sunflower or sea buckthorn), and different concentrations of beeswax (1 or 3%) were investigated. Concentrations were calculated based on the total mass of the emulsion. The preparation methodology is presented in Figure 1. Aqueous and oily phases were heated to 70–80 °C and mixed and homogenized using different times (1, 3, or 5 min) and rotation speeds (15,000 or 20,000 rpm) (T25 digital ULTRA-TURRAX disperser, IKA Werke, Staufen, Germany). After evaluating oily droplet sizes of prepared emulsions, 3 min and 20,000 rpm homogenization parameters were selected as optimal for preparing other samples. Afterward, they were frozen (−20 °C) on glass plates and freeze-dried (−55 °C, 5 Pa, 24 h) (α 1–2 LD plus lyophilizator, Martin Christ, Osterode am Harz, Germany).

**Figure 1:**
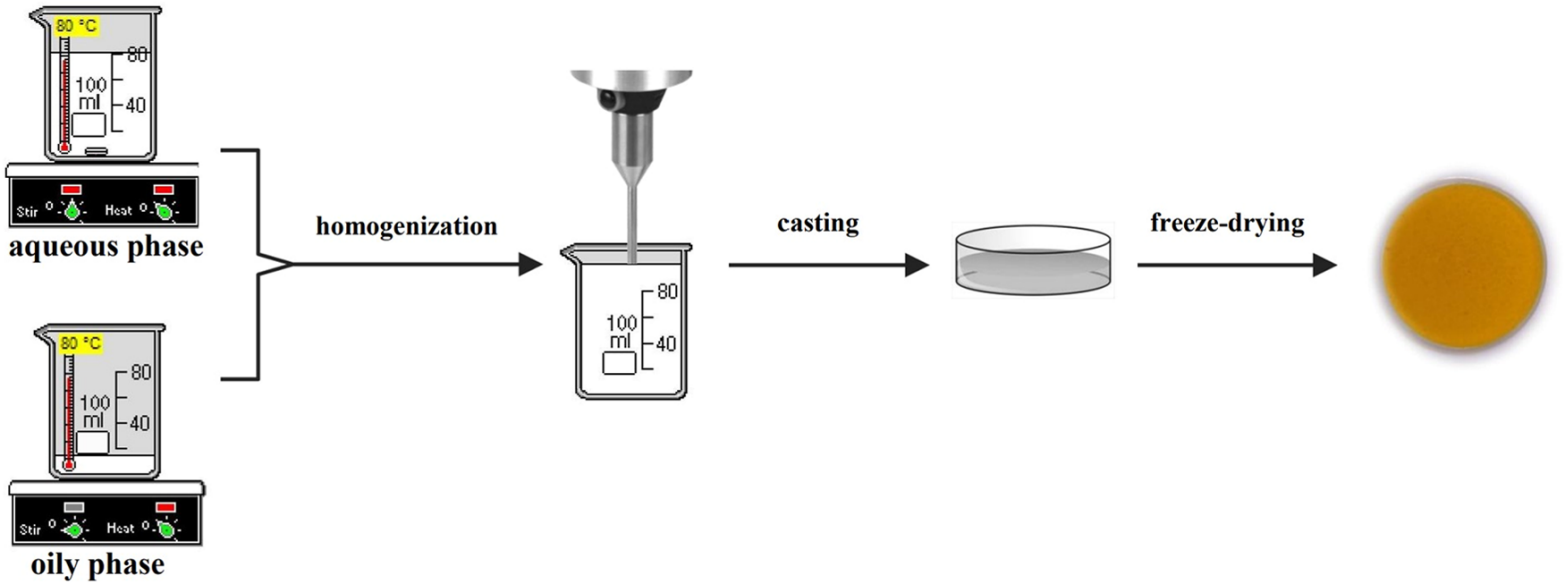
Preparation method for freeze-dried emulsions.

**Table 1:** Composition of the Prepared Materials^a^

		aqueous phase			oily phase		
sample	oily/aqueous phase ratio	polymers		cryoprotectant	oil	emulsifier	wax
1% WPI + 2% ALG + 1% G + SO_5/95	5/95	1% of WPI	2% of ALG	1% of G	SO	1% of Span 80	-
1% WPI + 2% ALG + 1% G + SO_10/90	10/90	1% of WPI	2% of ALG	1% of G	SO	1% of Span 80	-
1% WPI + 2% ALG + 1% G + SO_15/85	15/85	1% of WPI	2% of ALG	1% of G	SO	1% of Span 80	-
3% WPI + 2% ALG + 1% G + SO_5/95	5/95	3% of WPI	2% of ALG	1% of G	SO	1% of Span 80	-
3% WPI + 2% ALG + 1% G + SO_10/90	10/90	3% of WPI	2% of ALG	1% of G	SO	1% of Span 80	-
3% WPI + 2% ALG + 1% G + SO_15/85	15/85	3% of WPI	2% of ALG	1% of G	SO	1% of Span 80	-
3% WPI + 2% ALG + SO_10/90	10/90	3% of WPI	2% of ALG	-	SO	1% of Span 80	-
3% WPI + 2% ALG + 3% G + SO_10/90	10/90	3% of WPI	2% of ALG	3% of G	SO	1% of Span 80	-
3% WPI + 2% ALG + 1% PG + SO_10/90	10/90	3% of WPI	2% of ALG	1% of PG	SO	1% of Span 80	-
3% WPI + 2% ALG + 3% PG + SO_10/90	10/90	3% of WPI	2% of ALG	3% of PG	SO	1% of Span 80	-
3% WPI + 2% ALG + 1% S + SO_10/90	10/90	3% of WPI	2% of ALG	1% of S	SO	1% of Span 80	-
3% WPI + 2% ALG + 3% S + SO_10/90	10/90	3% of WPI	2% of ALG	3% of S	SO	1% of Span 80	-
3% WPI + 2% ALG + 1% M + SO_10/90	10/90	3% of WPI	2% of ALG	1% of M	SO	1% of Span 80	-
3% WPI + 2% ALG + 3% M + SO_10/90	10/90	3% of WPI	2% of ALG	3% of M	SO	1% of Span 80	-
3% WPI + 2% ALG + 1% T + SO_10/90	10/90	3% of WPI	2% of ALG	1% of T	SO	1% of Span 80	-
3% WPI + 2% ALG + 3% T + SO_10/90	10/90	3% of WPI	2% of ALG	3% of T	SO	1% of Span 80	-
3% WPI + 2% ALG + 1% G + SBO_10/90	10/90	3% of WPI	2% of ALG	1% of G	SBO	1% of Span 80	-
3% WPI + 2% ALG + 1% G + SO + 1% B_10/90	10/90	3% of WPI	2% of ALG	1% of G	SO	1% of Span 80	1% of B
3% WPI + 2% ALG + 1% G + SBO+ 1% B_10/90	10/90	3% of WPI	2% of ALG	1% of G	SBO	1% of Span 80	1% of B
3% WPI + 2% ALG + 1% G + SO + 3% B_10/90	10/90	3% of WPI	2% of ALG	1% of G	SO	1% of Span 80	3% of B
3% WPI + 2% ALG + 1% G + SBO + 3% B_10/90	10/90	3% of WPI	2% of ALG	1% of G	SBO	1% of Span 80	3% of B

a WPI: Whey protein isolate; ALG: sodium alginate; G: glycerin; PG: propylene glycol; S: sorbitol; M: mannitol; T: trehalose; SO: sunflower oil; SBO: sea buckthorn oil; B: beeswax.

### 2.3 Characterization of the Materials

#### 2.3.1 Emulsion Droplet Size Distribution

The analysis of droplet size distributions of emulsions based on whey protein isolate, sodium alginate, cryoprotectants (glycerin, propylene glycol, sorbitol, mannitol, and trehalose), and lipids (sunflower oil, sea buckthorn oil, and beeswax) were carried out using a laser diffraction particle size analyzer (SALD-2300 with the SALD-MS23 sampler, Shimadzu, Kyoto, Japan). A small amount of emulsions was added to the sampler’s dispersion bath containing distilled water. During circulation, the oily emulsion droplets were dispersed between the flow cell and the dispersion bath and irradiated with a laser beam in the measurement unit. The light intensity distribution of scattered light was used to calculate the oil droplet size distribution using Wing SALD II software (ver. 3.1.0, Shimadzu, Kyoto, Japan). The width of the droplet size distribution (span) was calculated using eq 1

1 $$\mathrm{span}=\left(X_{90}-X_{10}\right)/X_{50}$$

where *X*_10_, *X*_50_, and *X*_90_ represent the volume percentages of oil droplets (10, 50, and 90% undersize, respectively).

#### 2.3.2 Imaging

Scanning electron microscopy (SEM) imaging (Quanta 3D FEG scanning electron microscope, Quorum Technologies, Lewes, U.K.) was applied in order to evaluate the structures and cross-sections of the obtained porous materials. A thin layer of gold and palladium (SC7620 mini sputter coater/glow discharge system, Quorum Technologies, Lewels, U.K.) was spread on the surfaces of the freeze-dried emulsions before the analysis.

#### 2.3.3 Mechanical Properties

A mechanical testing machine (Shimadzu EZ-Test EZ-SX, Kyoto, Japan) fitted with a 50 N load cell was used to investigate the freeze-dried emulsions’ mechanical properties. The compression results (5 mm/min compression speed) of seven cylindrical samples of each material type with a diameter of 10 mm were recorded using the Trapezium X Texture program (version 1.4.5.). The Young’s modulus and compressive maximum force were calculated from the stress–strain curves.

#### 2.3.4 Porosity and Density Measurements

The liquid displacement method was employed in order to perform porosity (ε) and density (*d*) measurements of porous matrices. Isopropanol was selected as a nonsolvent of used polymers.^33^ The graduated cylinder was filled with isopropanol (*V*_1_). Weighed materials (*W*) were put into the graduated cylinder and left for 5 min. Subsequently, the isopropanol volume was noted (*V*_2_), and it was reread after careful removal of the materials (*V*_3_). Measurements of the porosity (eq 2) and density (eq 3) of materials were carried out in triplicate and calculated as follows

2 $$\epsilon \;\left(\%\right)=\left(V_{1}-V_{3}\right)/\left(V_{2}-V_{3}\right)\cdot 100$$

3 $$d=W/\left(V_{2}-V_{3}\right)$$

#### 2.3.5 Residual Moisture Content

The residual moisture contents of freeze-dried emulsions based on WPI, sodium alginate, different cryoprotectants, and oily substances were assessed as the percentage of the water removed from the samples dried to the constant weight (eq 4). Weighed samples (1 cm × 1 cm) (*W*_w_) were dried at 105 °C for 24 h and then weighed again (*W*_d_). The measurements were carried out in triplicate and calculated as follows

4 $$\mathrm{MC}\;\left(\%\right)=\left(W_{w}-W_{d}\right)/W_{w}\cdot 100$$

#### 2.3.6 Statistical Analysis

The Past 4.09 program (PAleontological Statistics Software, Oslo, Norway) was used to carry out one-way ANOVA with Tukey’s pairwise analysis to compare results statistically. Data are shown as the mean ± SD for each experiment with *p*-values ≤0.05 considered significant.

## 3 Results and Discussion

### 3.1 Emulsion Droplet Size Distribution

In order to optimize the preparation method of emulsions, different times (1, 3, and 5 min) and speeds (15,000 and 20,000 rpm) of emulsion homogenization were examined. 3% WPI + 2% ALG + 1% G + SO_10/90 was chosen as an exemplary emulsion. The emulsion droplet size distribution was expressed as the mean droplet size and span, which measures the breadth of the distribution.

The oily droplet size distribution charts and characteristics of these droplets are presented in Figure 2 and Table 2, respectively. Based on the obtained results, one can conclude that the size distributions of oily droplets in the obtained emulsions depend on the rotation speed of the homogenization of water and oily phases as well as the time of homogenization. The span ranged from 2.62 for the sample homogenized for 3 min at 20,000 rpm to 28.33 for the sample fabricated at 1 min and 15,000 rpm. The mean droplet size ranged from 2.02 to 7.53 μm with smaller mean droplet sizes and spans for samples homogenized at higher speeds (20,000 rpm instead of 15,000 rpm) regardless of the homogenization time. However, homogenization during 1, 3, and 5 min at 15,000 rpm created emulsions with larger mean droplet sizes and polydispersity, which may indicate incomplete homogenization. One can also conclude that all prepared samples were macroemulsions based on the diameters of the oily droplet sizes.

**Table 2:** Characteristics of 3% WPI + 2% ALG + 1% G + SO_10/90 Emulsion Droplets Depending on the Time (1, 3, and 5 min) and Speed (15,000 and 20,000 rpm) of Homogenization^a^

		droplet size (μm)			
sample (time and speed of homogenization)	mean droplet size (μm ± SD)	*X*_10_	*X*_50_	*X*_90_	span
1 min, 15,000 rpm	6.64 ± 0.79	0.82	3.81	108.69	28.33
1 min, 20,000 rpm	3.96 ± 0.59	0.84	2.72	28.90	10.33
3 min, 15,000 rpm	7.53 ± 0.68	0.93	10.88	50.89	4.59
3 min, 20,000 rpm	2.02 ± 0.34	0.79	1.88	5.72	2.62
5 min, 15,000 rpm	4.58 ± 0.65	0.84	2.89	44.09	14.99
5 min, 20,000 rpm	2.03 ± 0.35	0.76	1.86	6.20	2.92

a *X*_10_, *X*_50_, and *X*_90_ represent the volume percentages of droplets (10, 50, and 90% undersize, respectively).

**Figure 2:**
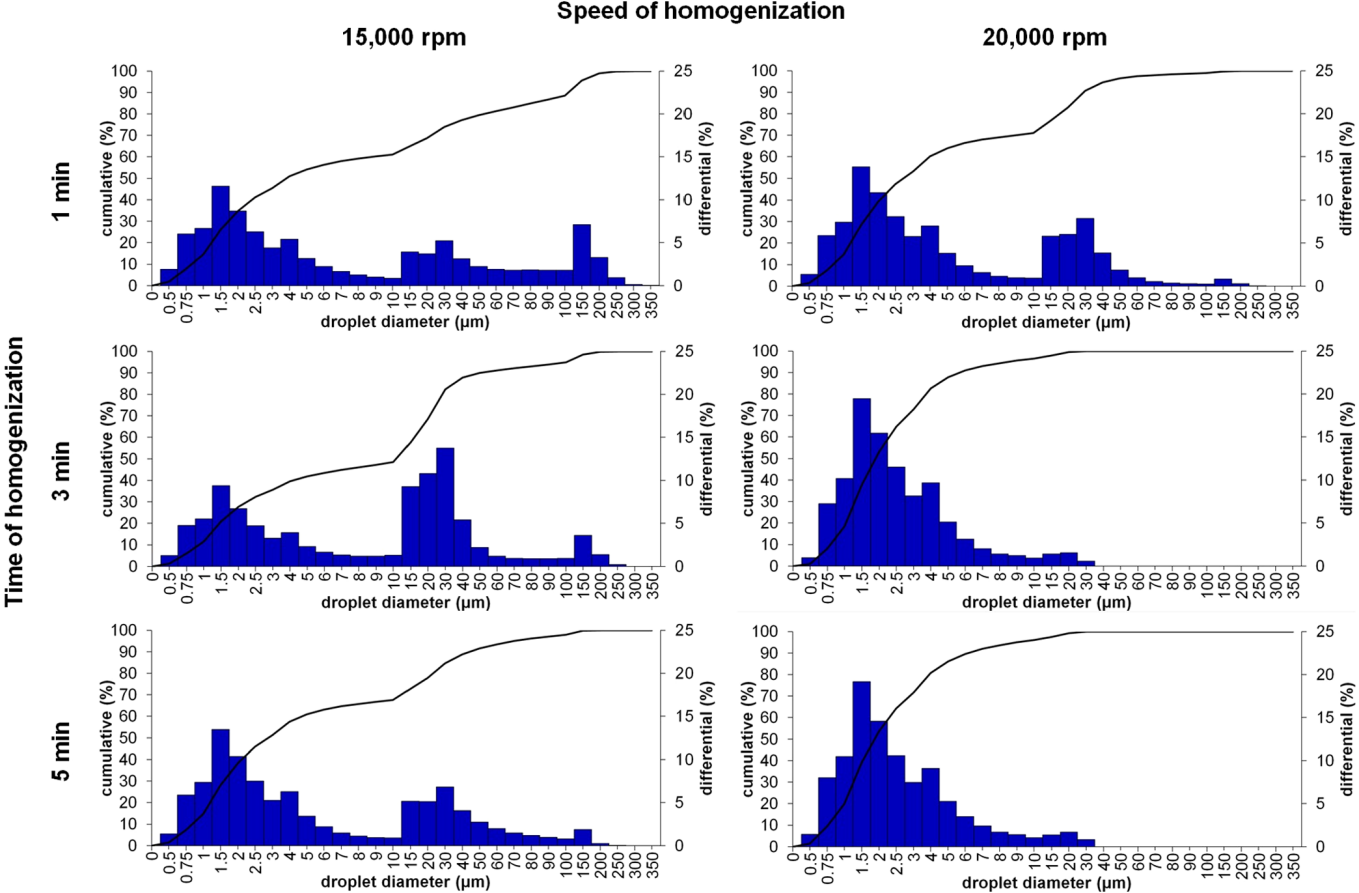
Droplet size distribution of the 3% WPI + 2% ALG + 1% G + SO_10/90 emulsion depending on the time (1, 3, and 5 min) and speed (15,000 and 20,000 rpm) of homogenization.

Distribution of the sizes of oil droplets within an emulsion is a crucial parameter impacting the stability, texture, appearance, functional properties, and performance of the emulsion, as well as the bioavailability of active ingredients.^34,35^ Uniformity, represented as a lower span and smaller size of the oily droplets, can improve the emulsion’s stability by increasing the emulsion’s surface area and thus reducing the coalescence or phase separation.^36,37^ Therefore, the most desired results were obtained for samples developed using a homogenizer for 3 and 5 min at 20,000 rpm. As presented in Figure 2, these samples presented very similar droplet diameters with a more narrow distribution. However, in order to prepare other samples, the parameters of time and speed were selected as 3 min and 20,000 rpm, respectively.

After establishing the time and speed of homogenization, different oily-to-aqueous phase ratios (5/95, 10/90, and 15/85) of samples containing 1 or 3% WPI, 2% sodium alginate, 1% glycerin in the aqueous phase, and sunflower oil with Span 80 in the oily phase were investigated. As shown in Figure 3, the oily droplet size distribution differed depending on the amount of the oily phase in the system and the amount of whey protein isolate in the aqueous phase. For materials developed with 1% WPI, the amount of oily phase droplets with larger diameters was lower than that of samples containing 3% WPI at 5/95 and 15/85 oily-to-aqueous phase ratios. This observation is also noted in Table 3, which contains the characteristics of these droplets. Higher spans and mean droplet sizes were noted for samples containing 3% WPI in 5/95 and 15/85 oily-to-aqueous phase ratios. For samples containing 1% WPI, the span was from 4.10 to 7.15, and the mean droplet size was from 2.28 to 3.21 μm, whereas for materials with 3% WPI at 5/95 and 15/85 mixing ratios, the span was from 9.58 to 10.30 and the mean droplet size was from 3.65 to 5.48 μm (an exception was the 3% WPI + 2% ALG + 1% G + SO_10/90 sample with the lowest droplet diameter size and span). Based on the results, the 10/90 oily-to-aqueous phase ratio was chosen as the optimal amount of the oily phase in the emulsion system. Larger oily droplet diameters and a wider size distribution were noted for samples containing higher amounts of WPI, which could be associated with the hydrophobic areas in WPI.^38^ Due to the amino acid sequences and three-dimensional structures of β-lactoglobulin, α-lactalbumin, serum albumin, and immunoglobulins, WPI consists of hydrophobic and hydrophilic regions. Hydrophobic areas are correlated with nonpolar side chains of amino acids such as leucine, valine, and phenylalanine. In contrast, hydrophilic regions are linked with polar or charged side chains of serine, glutamine, and lysine.^39^ The balance between these two regions can lead to the additional stabilization of emulsions since the hydrophobic parts adsorb into the oil droplets, preventing their coalescence and aggregation.^40,41^ Furthermore, the larger oil content was associated with the increase in polydispersity of the emulsion and the formation of larger emulsion droplet sizes.^42,43^

**Figure 3:**
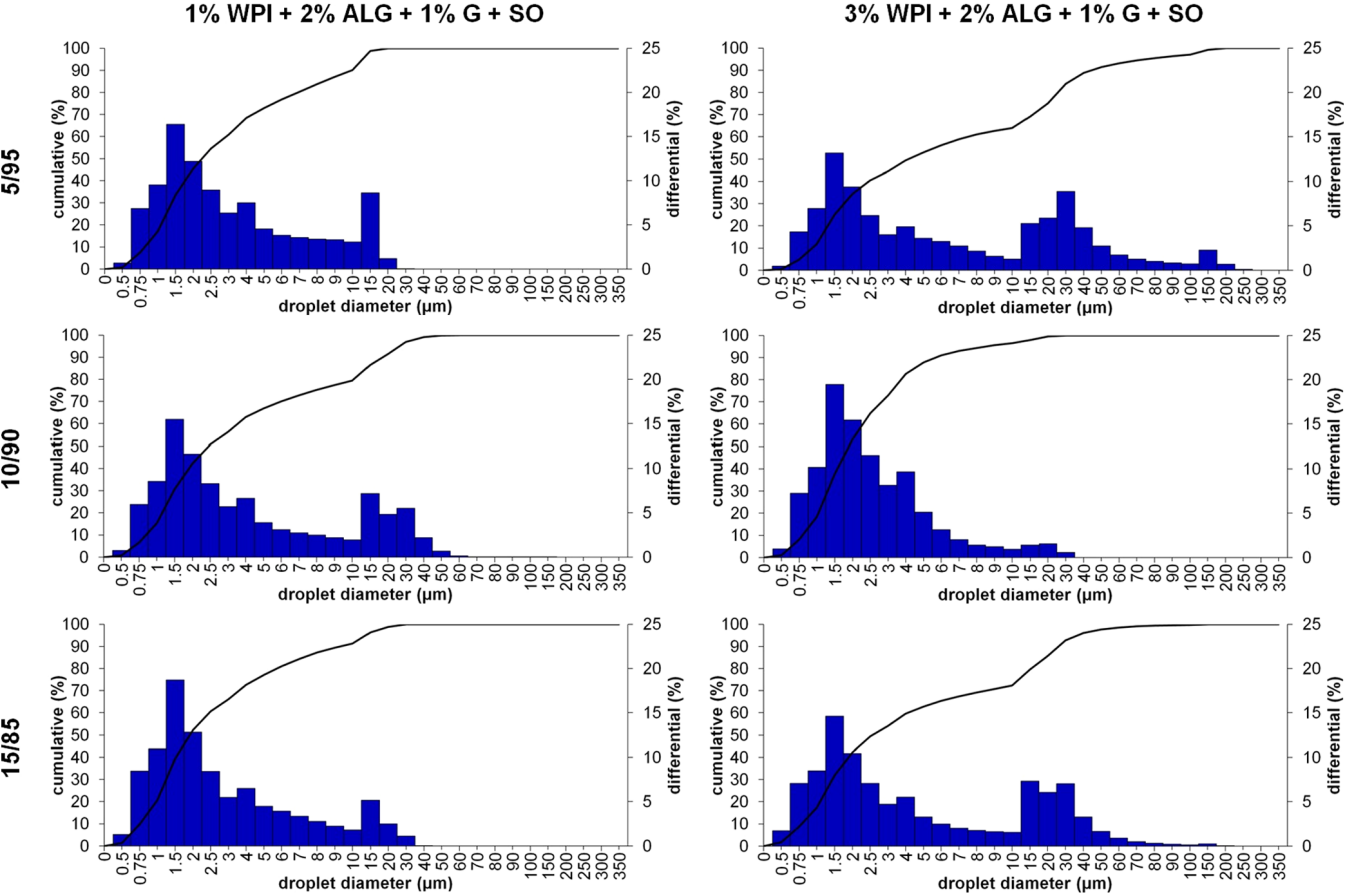
Droplet size distribution of samples containing 1 and 3% WPI, 2% sodium alginate, 1% glycerin, and sunflower oil with Span 80 at different oily-to-aqueous phase ratios: 5/95, 10/90, and 15/85.

**Table 3:** Characteristics of Emulsion Droplets in Samples Based on Biopolymers (Sodium Alginate and Whey Protein Isolate), Cryoprotectants (Glycerin, Propylene Glycol, Sorbitol, Mannitol, and Trehalose), Oils (Sunflower Oil and Sea Buckthorn Oil), Beeswax, and Emulsifiers (Span 80), As Well As Different Oily-to-Aqueous Phase Ratios (5/95, 10/90, and 15/85)^a^

		droplet size (μm)			
sample	mean droplet size (μm ± SD)	*X*_10_	*X*_50_	*X*_90_	span
1% WPI + 2% ALG + 1% G + SO_5/95	2.55 ± 0.40	0.81	2.23	9.95	4.10
1% WPI + 2% ALG + 1% G + SO_10/90	3.21 ± 0.50	0.84	2.46	18.40	7.15
1% WPI + 2% ALG + 1% G + SO_15/85	2.28 ± 0.41	0.75	1.90	9.36	4.53
3% WPI + 2% ALG + 1% G + SO_5/95	5.48 ± 0.65	0.94	4.17	43.94	10.30
3% WPI + 2% ALG + 1% G + SO_10/90	2.02 ± 0.34	0.79	1.88	5.72	2.62
3% WPI + 2% ALG + 1% G + SO_15/85	3.65 ± 0.58	0.78	2.56	25.32	9.58
3% WPI + 2% ALG + SO_10/90	2.39 ± 0.35	0.94	2.25	8.44	3.33
3% WPI + 2% ALG + 3% G + SO_10/90	3.30 ± 0.56	1.08	2.54	23.73	8.91
3% WPI + 2% ALG + 1% PG + SO_10/90	2.23 ± 0.39	0.78	1.94	8.61	4.04
3% WPI + 2% ALG + 3% PG + SO_10/90	3.66 ± 0.56	0.86	2.55	25.69	9.75
3% WPI + 2% ALG + 1% S + SO_10/90	4.01 ± 0.59	0.92	2.74	32.12	11.38
3% WPI + 2% ALG + 3% S + SO_10/90	6.00 ± 0.65	0.99	4.24	44.46	10.24
3% WPI + 2% ALG + 1% M + SO_10/90	3.95 ± 0.61	0.82	2.67	32.52	11.89
3% WPI + 2% ALG + 3% M + SO_10/90	3.29 ± 0.52	0.86	2.46	21.69	8.48
3% WPI + 2% ALG + 1% T + SO_10/90	3.68 ± 0.54	0.88	2.69	24.56	8.82
3% WPI + 2% ALG + 3% T + SO_10/90	5.05 ± 0.66	0.90	3.30	47.56	14.16
3% WPI + 2% ALG + 1% G + SBO_10/90	4.01 ± 0.60	0.85	2.70	31.16	11.23
3% WPI + 2% ALG + 1% G + SO + 1% B_10/90	21.56 ± 0.63	1.82	32.37	101.88	3.09
3% WPI + 2% ALG + 1% G + SBO + 1% B_10/90	20.02 ± 0.65	1.63	31.16	101.30	3.20
3% WPI + 2% ALG + 1% G + SO + 3% B_10/90	11.60 ± 0.69	1.14	17.99	75.89	4.15
3% WPI + 2% ALG + 1% G + SBO + 3% B_10/90	11.40 ± 0.67	1.18	16.97	75.49	4.38

a *X*_10_, *X*_50_, and *X*_90_ represent the volume percentages of droplets (10, 50, and 90% undersize, respectively).

Samples containing 3% WPI, 2% sodium alginate in an aqueous phase, and sunflower oil with the emulsifier in the oily phase were modified with different types and concentrations of cryoprotectants: glycerin, propylene glycol, sorbitol, mannitol, and trehalose. Figure 4 presents their oily droplet size distribution. The size distribution of the dispersed phase droplets in the emulsion continuous phase slightly depended on the presence of cryoprotectants in the sample composition. Samples containing 1% of the addition of the cryoprotectant had a mean droplet diameter of 2.02–4.01 μm and a span value from 2.62 to 11.89, whereas 3% of cryoprotectant addition led to a mean droplet size from 3.29 to 6.00 μm and a span value from 8.48 to 14.16 (Table 3). The sample without cryoprotectants in the material composition had a 2.39 μm mean droplet size and a 3.33 span value. Higher characteristics of the oily droplets in samples containing higher amounts of cryoprotectants might be connected with their effects on the emulsions’ viscosity and interfacial properties.^44^ Increasing the viscosity of the continuous phase may result in a restricted movement of the oil droplets and emulsifier effectiveness.^45^ The bimodal distribution of emulsion droplets may also reflect multiple droplet populations.

**Figure 4:**
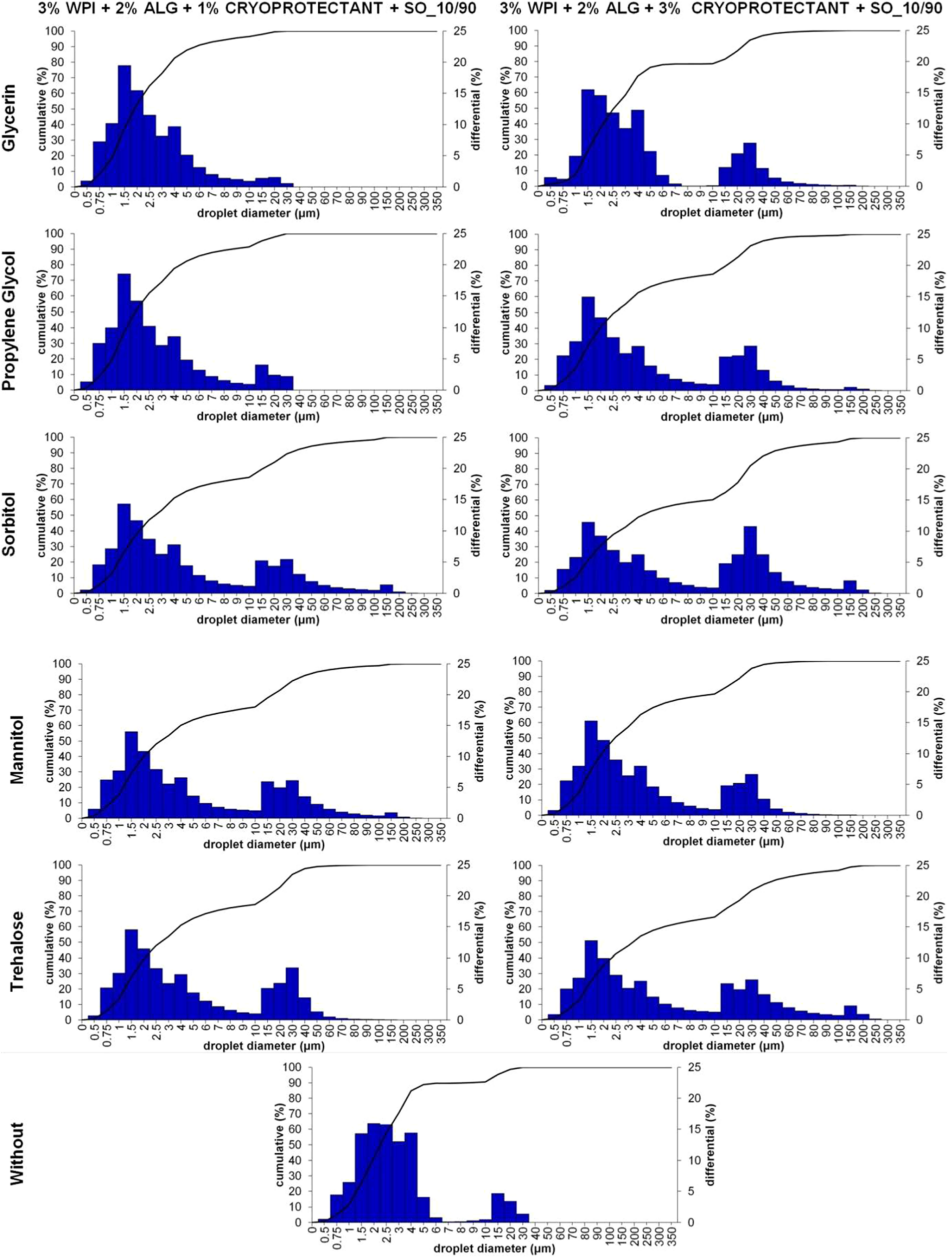
Droplet size distribution of samples containing 3% WPI, 2% sodium alginate, sunflower oil, and Span 80 with and without the addition of 1 or 3% of cryoprotectants (glycerin, propylene glycol, sorbitol, mannitol, and trehalose) with a 10/90 oily-to-aqueous phase ratio.

Figure 5 presents the oily droplet size distribution of samples containing the same aqueous phase: 3% WPI, 2% sodium alginate, and 1% glycerin, with an alteration regarding the oily phase. Materials were fabricated using sunflower oil or sea buckthorn oil with the addition of 1 or 3% beeswax. One can see a shift in the droplet size distribution. After the addition of beeswax into the emulsion composition, the number of droplets with higher diameters was larger than that of the smaller droplets. Therefore, the addition of beeswax led to a significant rise in the mean droplet size from 2.02–4.01 to 11.40–21.56 μm depending on the amount of beeswax; a lower amount of beeswax caused a higher rise in oily droplet sizes (Table 3). The span value of droplets was lower for samples containing beeswax and was from 3.09 to 4.38, whereas the sample with sea buckthorn oil and emulsifiers in the oily phase had a span value of 11.23. Therefore, despite a narrow distribution of oily droplets containing beeswax, their diameter was higher than for samples without beeswax. Higher oily droplet diameters may be connected with a higher density and viscosity of the oily phase after the addition of beeswax, which also led to a more homogeneous internal network.^46,47^ Samples with broader or bimodal droplet size distributions may demonstrate reduced homogeneity. However, the freeze-drying process can induce or contribute to structural rearrangements within the emulsion matrix, potentially leading to partial homogenization and modification of the physicochemical properties.

**Figure 5:**
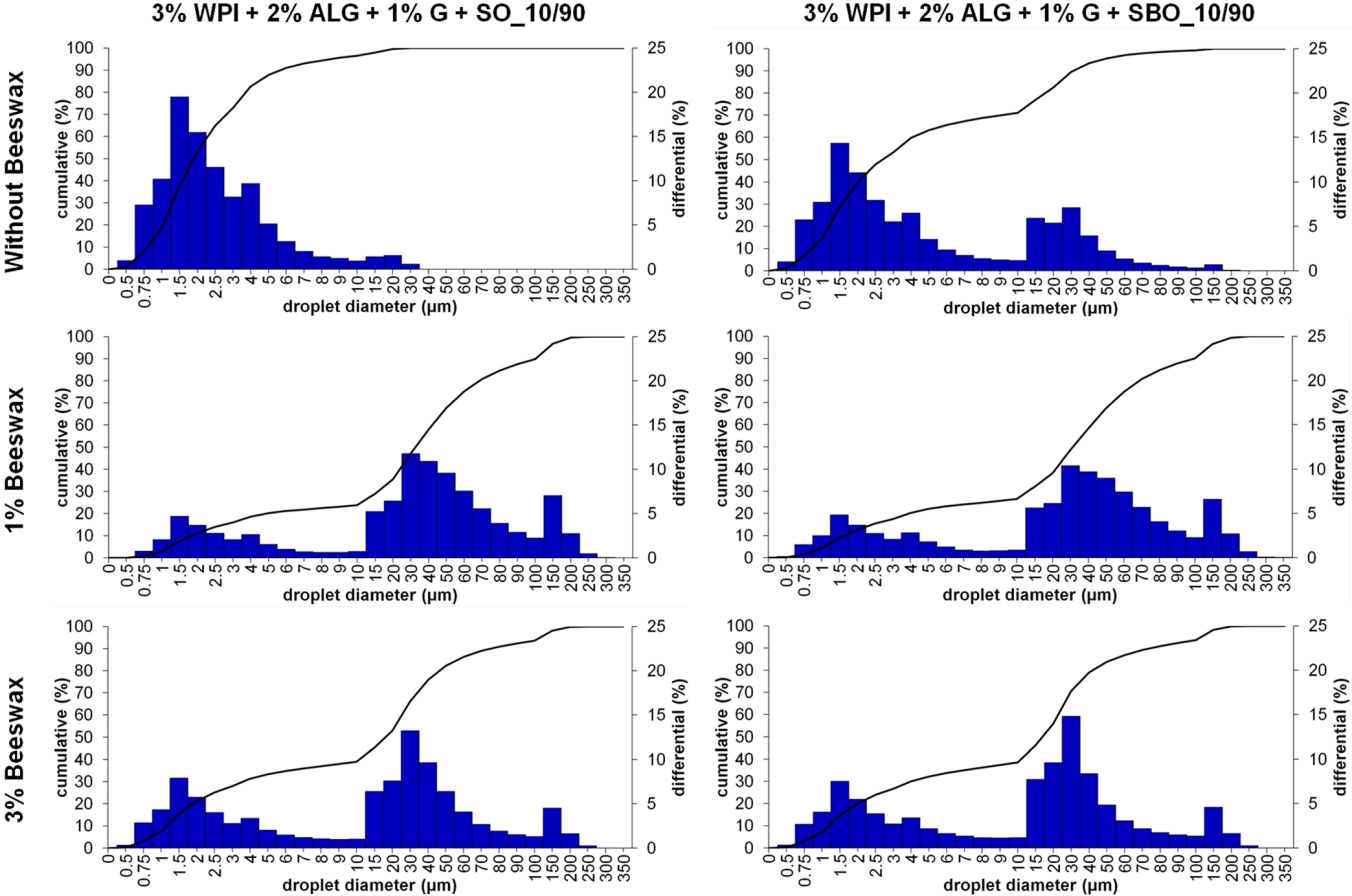
Droplet size distribution of samples containing 3% WPI, 2% sodium alginate, 1% glycerin in the aqueous phase, and sunflower oil or sea buckthorn oil with 1 of 3% addition of beeswax and emulsifiers (Span 80) in the oily phase with a 10/90 oily-to-aqueous phase ratio.

### 3.2 Appearance and Structure of Materials

Freeze-drying of prepared emulsions based on biopolymers (sodium alginate and whey protein isolate), cryoprotectants (glycerin, propylene glycol, sorbitol, mannitol, and trehalose), oils (sunflower oil and sea buckthorn oil), beeswax, and emulsifiers (Span 80) resulted in the fabrication of three-dimensional matrices. Pictures and SEM images showing the structure of obtained materials are presented in Figure 6. FD emulsions were soft and spongy; however, some compositions tend to be slightly more hard, brittle, and rigid. SEM images revealed that all matrices exhibited a complex internal structure featuring irregular interconnected macropores. However, several samples such as 3% WPI + 2% ALG + SO_10/90, 3% WPI + 2% ALG + 3% S + SO_10/90, 3% WPI + 2% ALG + 1% T + SO_10/90, 3% WPI + 2% ALG + 1% G + SBO_10/90, and 3% WPI + 2% ALG + 1% G + SO + 3% B_10/90 had a more linear and irregularly arranged structure with a longitudinal alignment of pores throughout the length of the sponges. These samples also appear to contain more lamellar or channel-like structures formed by the alignment of pores in a single direction. Samples containing sunflower oil as the basis of the oily phase had a whitish color, whereas samples with sea buckthorn oil had a vibrant orange color due to the intense color of this oil.

**Figure 6:**
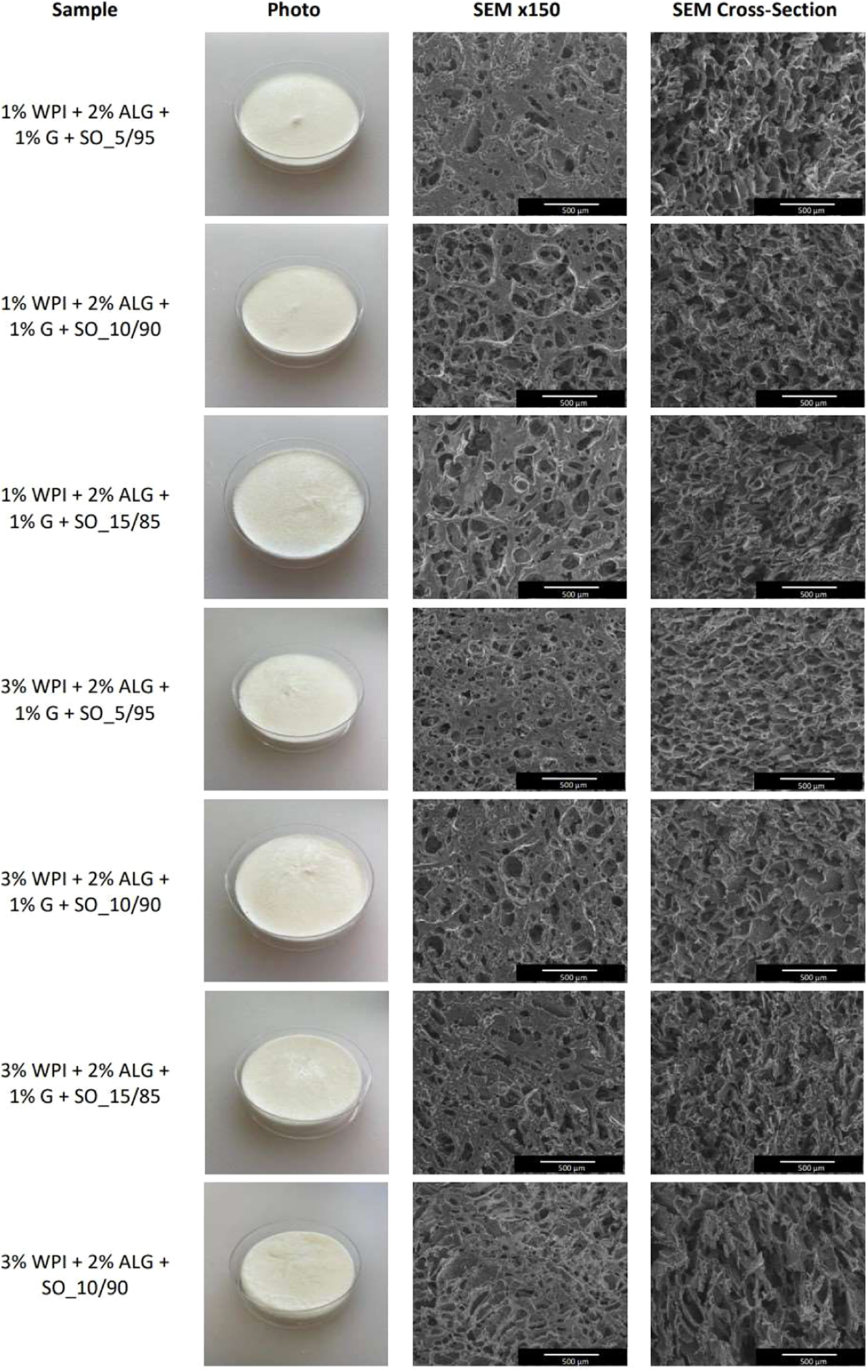

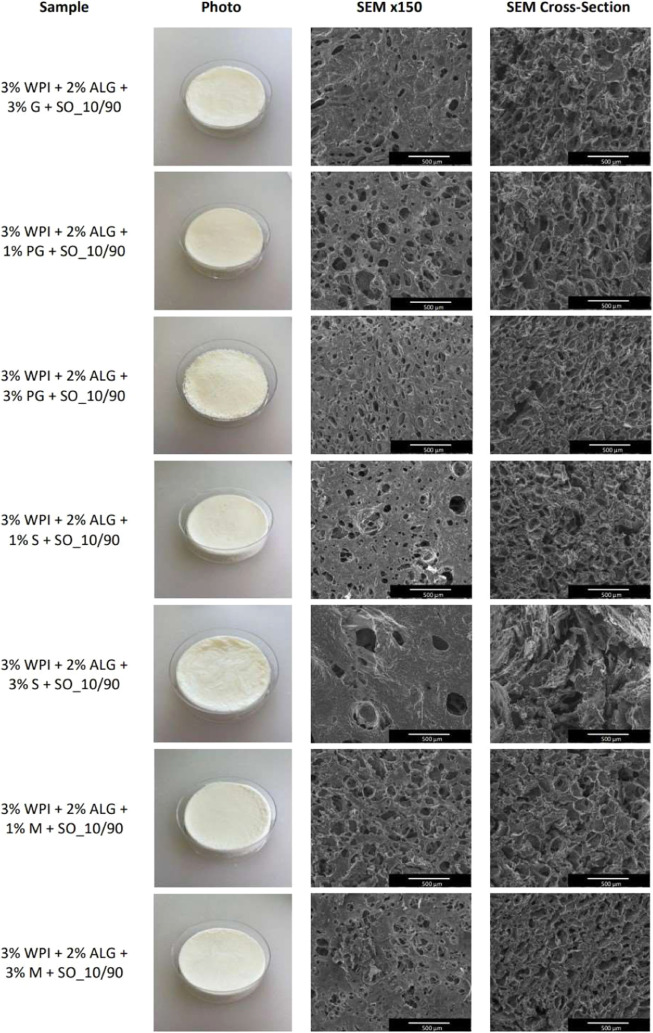
Pictures of obtained materials (the diameter of the container is 60 mm) and SEM images of their structures at a magnification of ×150 (scale bar = 500 μm) and cross-sections at a magnification of ×150 (scale bar = 500 μm).

Porous structure formation is influenced by the nucleation of ice grains within the polymer network, which are replaced by macropores during sublimation. However, during the freezing of samples, some mechanical stress may occur, including aggregation of the oily phase, which could potentially destabilize the emulsified system. To avoid such damage, cryoprotectants such as glycerin, propylene glycol, sorbitol, mannitol, or trehalose are employed due to their help in the prevention of ice crystallization. Do Vale Morais et al. developed a freeze-dried microemulsion for drug delivery purposes, evaluating different types and concentrations of cryoprotectants (mannitol, glucose, lactose, sorbitol, and maltose).^48^ Their results indicated maltose as the most effective cryoprotectant. Iyer et al. examined the effect of the addition of sucrose, trehalose, and mannitol to freeze-dried emulsions as vaccine drug products.^21^ They concluded that sucrose appeared to be the most effective in the preservation of the droplet size. Nevertheless, in another study, mannitol was superior to trehalose, lactose, and glycine as a protective agent during evening primrose oil microemulsion freeze-drying.^49^

### 3.3 Mechanical Properties

Mechanical properties during compression of prepared freeze-dried emulsions were evaluated using the Young’s modulus and compressive maximum force (Table 4). Mechanical properties influence the product handling, storage durability, and user experience. Freeze-dried emulsions with higher mechanical strength resist crumbling and maintain structural integrity during transport while still being soft enough to be rehydrated and applied without difficulty. The Young’s modulus and compressive maximum force significantly depended on the composition of materials: the oily-to-aqueous phase ratio, concentration of WPI, presence and concentration of cryoprotectants, alteration of the oily phase-oil type, and the addition of beeswax. The values of the compressive maximum force differed from ∼4 N (for sample 1% WPI + 2% ALG + 1% G + SO_5/95) to ∼28 N (for sample 3% WPI + 2% ALG + 3% T + SO_10/90) with similar dependencies as the Young’s modulus. The Young’s modulus of samples varied from ∼240 kPa to ∼1.7 MPa. The amount of WPI in the sample with a 5/95 oily-to-aqueous phase ratio played a crucial role, namely, for the 1% WPI + 2% ALG + 1% G + SO_5/95 sample, the Young’s modulus was the lowest (240 kPa) and for the 3% WPI + 2% ALG + 1% G + SO_5/95 sample, it was significantly higher (1.3 MPa). In other mixing ratios, the content of WPI did not notably alter the Young’s modulus; however, its value was higher for samples with a 10/90 oily-to-aqueous ratio (∼600–670 kPa) compared to a 15/85 ratio (∼330–350 kPa). It has been found that increasing the WPI concentration increases the mechanical strength of materials up to a point, after which it decreases.^50^ A higher WPI concentration in freeze-dried materials tends to form a denser and more interconnected network through enhanced hydrogen bonding and van der Waals forces, contributing to their mechanical stiffness.^51^ The oil-to-water ratio also influences the material’s ability to resist deformation due to the plasticizing effect of more oil in the matrix composition.^52,53^ The oil might also act as a lubricant between the protein molecules, decreasing the material’s resistance to applied stress and potentially disrupting the formation of a strong biopolymeric network, resulting in a more flexible structure.

**Table 4:** Mechanical Properties of Prepared Freeze-Dried Emulsion Matrices Based on Biopolymers (Sodium Alginate and Whey Protein Isolate), Cryoprotectants (Glycerin, Propylene Glycol, Sorbitol, Mannitol, and Trehalose), Oils (Sunflower Oil and Sea Buckthorn Oil), Beeswax, and Emulsifiers (Span 80), As Well As Different Oily-to-Aqueous Phase Ratios (5/95, 10/90, and 15/85) during Compression

sample	Young’s modulus (kPa)	compressive maximum force (N)
1% WPI + 2% ALG + 1% G + SO_5/95	239.5 ± 18.3	4.02 ± 0.24
1% WPI + 2% ALG + 1% G + SO_10/90	598.1 ± 62.9	9.04 ± 0.25
1% WPI + 2% ALG + 1% G + SO_15/85	350.1 ± 57.6	6.69 ± 1.19
3% WPI + 2% ALG + 1% G + SO_5/95	1296.3 ± 168.3	12.85 ± 2.29
3% WPI + 2% ALG + 1% G + SO_10/90	671.2 ± 87.5	11.90 ± 0.88
3% WPI + 2% ALG + 1% G + SO_15/85	331.2 ± 37.3	8.76 ± 0.93
3% WPI + 2% ALG + SO_10/90	509.5 ± 28.4	10.91 ± 0.73
3% WPI + 2% ALG + 3% G + SO_10/90	326.0 ± 38.8	8.22 ± 0.72
3% WPI + 2% ALG + 1% PG + SO_10/90	1699.4 ± 197.4	19.46 ± 0.32
3% WPI + 2% ALG + 3% PG + SO_10/90	589.5 ± 80.1	18.70 ± 0.74
3% WPI + 2% ALG + 1% S + SO_10/90	1122.7 ± 122.4	21.67 ± 1.37
3% WPI + 2% ALG + 3% S + SO_10/90	349.2 ± 17.0	17.18 ± 3.51
3% WPI + 2% ALG + 1% M + SO_10/90	1446.3 ± 137.2	19.74 ± 2.41
3% WPI + 2% ALG + 3% M + SO_10/90	1154.5 ± 143.9	19.95 ± 2.88
3% WPI + 2% ALG + 1% T + SO_10/90	1545.4 ± 95.5	14.96 ± 0.66
3% WPI + 2% ALG + 3% T + SO_10/90	1359.6 ± 207.2	28.20 ± 1.09
3% WPI + 2% ALG + 1% G + SBO_10/90	705.9 ± 84.1	11.86 ± 0.54
3% WPI + 2% ALG + 1% G + SO + 1% B_10/90	906.4 ± 63.5	10.67 ± 0.70
3% WPI + 2% ALG + 1% G + SBO + 1% B_10/90	844.6 ± 76.2	9.44 ± 0.48
3% WPI + 2% ALG + 1% G + SO + 3% B_10/90	1098.6 ± 102.8	13.76 ± 0.55
3% WPI + 2% ALG + 1% G + SBO + 3% B_10/90	960.8 ± 145.1	11.69 ± 0.68

Materials without cryoprotectants in their composition exhibited a Young’s modulus of ∼510 kPa. Furthermore, the presence of different cryoprotectants played a crucial role in the materials’ mechanical resistance to compression. This parameter was also higher for samples containing 1% cryoprotectant addition. The highest Young’s modulus was noted for 1% addition of propylene glycol (∼1.7 MPa), 1 and 3% addition of mannitol (∼1.4 and ∼1.2 MPa, respectively) and trehalose (∼1.5 and ∼1.4 MPa, respectively), and 1% addition of sorbitol (∼1.1 MPa). Lower values of the Young’s modulus exhibited materials containing a 3% addition of glycerin (326 kPa), propylene glycol (∼590 kPa), and sorbitol (∼350 kPa). Significant differences in the Young’s modulus between the concentrations of cryoprotectants were recorded for glycerin, propylene glycol, and sorbitol. However, solely a 3% addition of glycerin and sorbitol decreased the Young’s modulus below the value noted for the sample without cryoprotectants. The higher Young’s modulus of samples is related to materials more resistant to deformation under stress. More stiff and rigid matrices are more likely to retain their shape under compression and will not deform easily under applied force, maintaining their structure. Cryoprotectants also work as plasticizers, which are added to increase flexibility and reduce the brittleness of freeze-dried matrices.^54^ Their influence on mechanical properties depends on their concentration, molecular structure, and, hence, formed interactions with other components of matrices.^55,56^ They reduce intermolecular interactions between the protein and polysaccharide molecules within the matrix. Differences in the mechanical properties of samples containing different protective agents might be attributed to their structure. Glycerin and propylene glycol act similarly due to the similar structure of small hydrophilic molecules with the ability to form hydrophilic bonds. Due to their low molecular weight, they can easily diffuse into polymer chains, disrupting polymer interactions by increasing chain mobility and thus decreasing stiffness. Glycerin has three hydroxyl groups, while propylene glycol has two, which can affect their plasticizing ability. Sorbitol and mannitol as sugar alcohols have six hydroxyl groups, presenting a larger, more complex molecule. Their multiple hydroxyl groups allow them to interact with emulsion constituents. However, mannitol tends to have a worse plasticizing ability than sorbitol due to its more crystalline structure. Trehalose is a disaccharide with nonreducing linkage by an α,α-1,1-glycosidic bond, which makes it more stable than other sugars. Owing to its structure, it provides a more cryoprotectant than plasticizing effect. Compared to glycerin and sorbitol, mannitol and trehalose tend to have a weaker plasticizing effect, especially in higher concentrations, contributing to reinforced polymer alignment and intermolecular spacing during freezing, resulting in a higher Young’s modulus.

The addition and further increase in the concentration of beeswax resulted in higher Young’s modulus values for both sunflower oil (∼0.9 and 1.1 MPa, respectively) and sea buckthorn oil (∼0.8 and 1 MPa, respectively). The rise in the Young’s modulus observed after incorporating beeswax into the matrix was likely due to its ability to blend with and bind within the biopolymer network.^57^ Beeswax may fill interstitial voids and reinforce the structural integrity of the matrix, leading to a denser and more cohesive material with enhanced mechanical resistance. However, the oil type did not lead to notably different values of the Young’s modulus; the sample with sunflower oil had 671.2 ± 87.5 kPa, while the material containing sea buckthorn oil had 705.9 ± 84.1 kPa.

### 3.4 Porosity and Density Measurements

The liquid displacement method was employed in order to examine the porosity (Figure 7) and density (Figure 8) of freeze-dried emulsions. Porosity influences the material’s ability to absorb and retain water upon rehydration rapidly, enabling efficient transformation back into the emulsion during application. Highly porous structures facilitate a quicker dissolution and better spreadability on the skin. Density, however, affects the product weight and packaging; lower-density formulations offer benefits in terms of lightweight packaging and reduced transport costs, aligning with sustainability goals.

**Figure 7:**
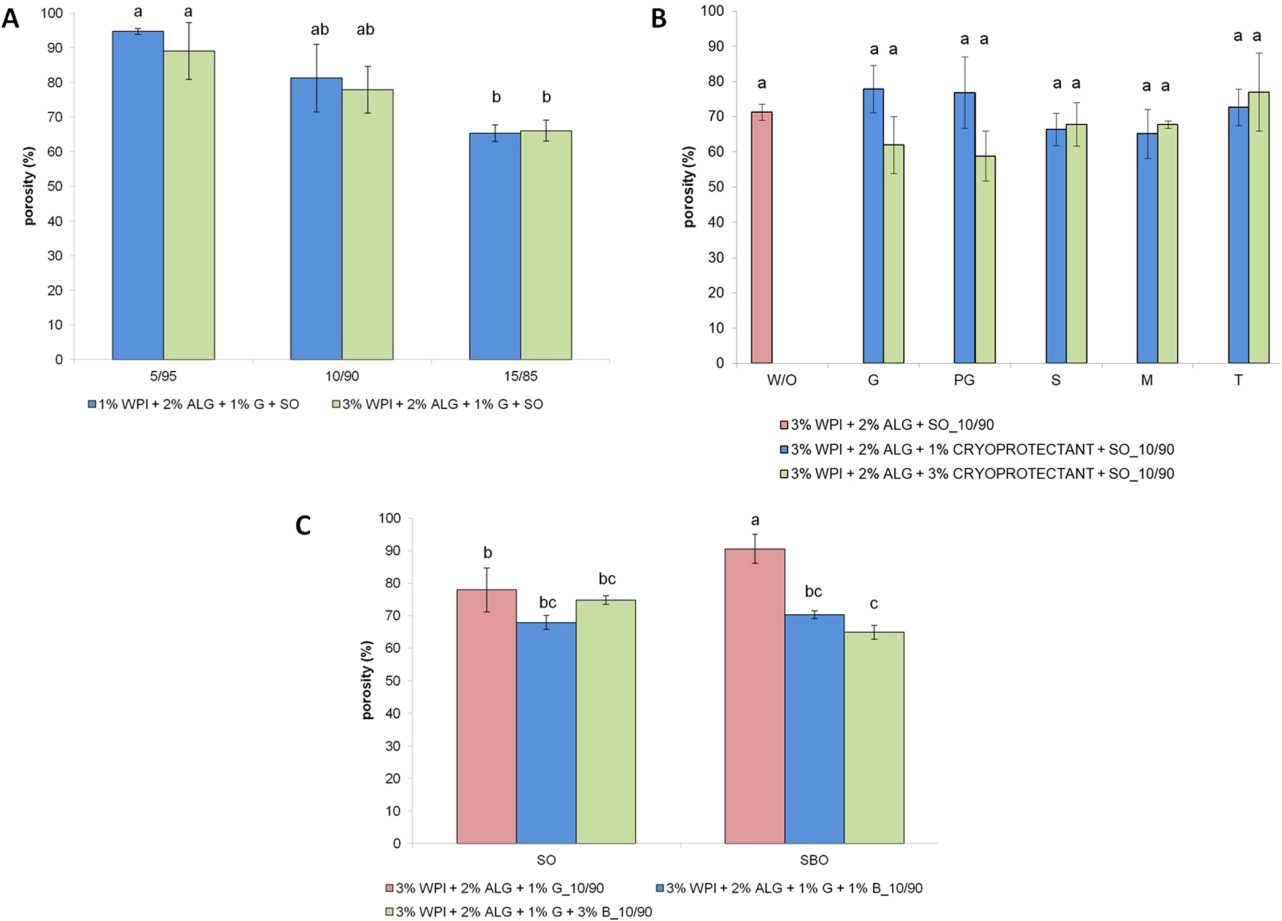
Porosity of freeze-dried emulsions with altering: (A) concentration of whey protein isolate at different oily-to-aqueous phase mixing ratios; (B) type and concentration of cryoprotectants (as well as a sample without the addition of cryoprotectants); and (C) type of oil and concentration of beeswax. Bars not sharing the same letter are significantly different (*p* ≤ 0.05).

**Figure 8:**
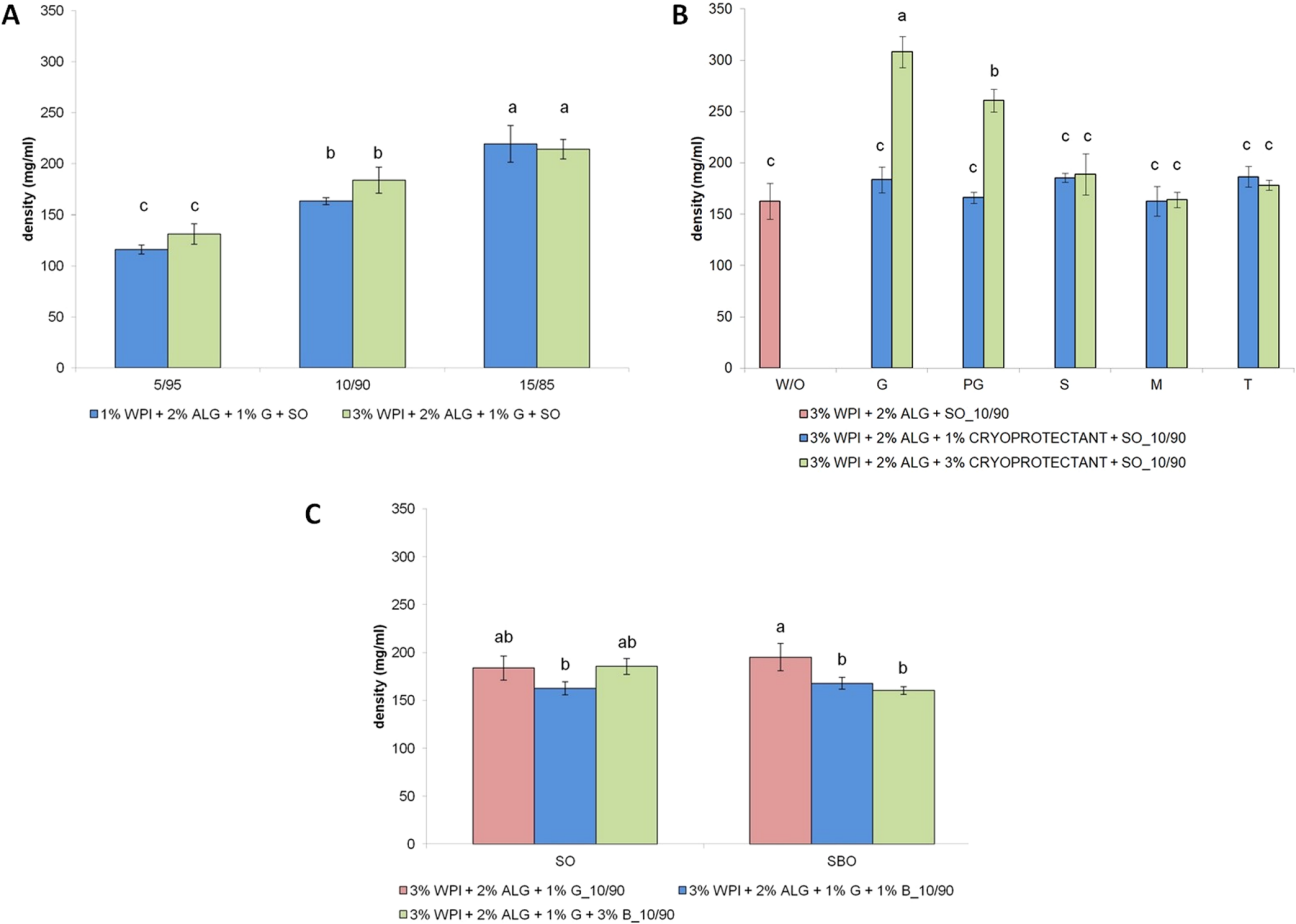
Density of freeze-dried emulsions with altering: (A) concentration of whey protein isolate at different oily-to-aqueous phase mixing ratios; (B) type and concentration of cryoprotectants (as well as a sample without the addition of cryoprotectants); and (C) type of oil and concentration of beeswax. Bars not sharing the same letter are significantly different (*p* ≤ 0.05).

The porosity varied from 59 to 95%. The porosity of prepared matrices significantly depends on the oily-to-aqueous phase mixing ratios; the higher the contribution of the oily phase in the material composition, the lower is their porosity (Figure 7A). The highest porosity (95%) was observed for the 1% WPI + 2% ALG + 1% G + SO_5/95 sample. Materials obtained with a 15/85 oily-to-aqueous phase ratio, regardless of the WPI concentration, had a porosity of ∼65%. Creating a porous structure depends on the formation of ice crystals that sublime during freeze-drying. Increasing the oil phase contribution reduces the pore amount, thus reducing the samples’ porosity. Samples with a higher aqueous phase content exhibited greater porosity due to more water available to form ice crystals, which later sublimated to leave behind larger or more numerous pores. Conversely, increasing the oil phase reduced porosity, as lipids do not sublime, thereby displacing water and decreasing the extent of pore formation. However, we did not observe significant differences in samples containing different whey protein concentrations.

The porosity of samples containing different types and concentrations of cryoprotectants and samples without the addition of cryoprotectants did not show statistically significant differences (Figure 7B). For these samples, the porosity ranged from 59 to 78%. This indicates that cryoprotectants were evenly distributed throughout the aqueous phase. This uniform distribution led to consistent water removal patterns and similar porosity of obtained samples despite differences in the cryoprotectant type and concentration.

Freeze-dried emulsions based on biopolymers and glycerin in the aqueous phase and sea buckthorn oil with an emulsifier in the oily phase showed 91% porosity (Figure 7C). The higher porosity observed for the sample containing sea buckthorn oil might be attributed to its higher polyunsaturated fatty acid content having more polar double bonds increasing their interaction with the aqueous phase. Modifying this sample with the addition of beeswax led to decreased material porosity (70% porosity for the sample containing 1% beeswax and 65% for the sample with 3% beeswax). The addition of beeswax into prepared samples created more dense and compact matrices due to its hydrophobicity, reducing the porosity. However, matrices containing sunflower oil as the basis of the oily phase did not exhibit significant differences in porosity values (68–78%).

The porosity of emulsion-based materials had been determined as 79–85% with decreased porosity for samples with a higher polymer concentration,^58^ 85–90% with decreasing values for samples with an increased contribution of the hydrophobic phase,^59^ and 88–98% depending on the polymer content and volume of the internal phase.^60^ Furthermore, sodium alginate-based matrices with polymer concentrations ranging from 4 to 16% had interconnected porosities of 83 to 58%, respectively, and total porosities ranging from 85 to 80%, respectively, with lower porosity for materials containing a higher polymer concentration.^61^ In comparison, Autissier et al. found that a decrease in freeze-drying pressure significantly increased the sample porosity from 33 to 68%.^62^

The density of prepared materials ranged from ∼116 to ∼308 mg/mL. The density of fabricated matrices did not significantly depend on the concentration of whey protein isolate (Figure 8A). Nonetheless, the higher the oily-to-aqueous phase mixing ratio, the higher the density of samples, from 115 to 131 and 214–219 mg/mL. Density results are consistent with those obtained for the porosity of matrices. As the oily phase did not sublime during freeze-drying, its higher contribution resulted in an increased density by reducing the porosity of the matrix. The proportion of the material that remained postdrying also increased, contributing to a higher solid mass per unit volume, thereby increasing the density.

The sample presenting the highest density contained a 3% addition of glycerin (∼308 mg/mL) and a 3% addition of propylene glycol (∼261 mg/mL) (Figure 8B). Glycerin and propylene glycol are more hygroscopic and have a stronger affinity for water molecules, allowing them to retain water in the matrix more effectively. This can contribute to these samples’ higher density and a higher residual moisture content. They could also penetrate and occupy intrapolymer spaces, especially at higher concentrations, reducing the extent of porous voids by limiting the expansion of ice crystals during freezing, hence increasing the density. Moreover, they are smaller and more flexible molecules than sorbitol, mannitol, and trehalose, providing easier integration into the matrix and increasing the samples’ density. Materials not modified with the addition of cryoprotectants and samples containing 1% glycerin and propylene glycol, 1 and 3% sorbitol, mannitol, and trehalose did not show statistically significant differences in density (∼163–189 mg/mL).

Materials containing sunflower oil did not exhibit differences in density after the addition of beeswax (∼162–185 mg/mL; Figure 8C), whereas freeze-dried emulsions containing sea buckthorn oil had a higher density (∼195 mg/mL) than samples modified with the addition of beeswax (∼160–168 mg/mL).

Our results are in line with those obtained by other research groups. It was established that materials based on high internal phase emulsions using freeze-drying, vacuum-drying, and heat-drying presented densities from 19 to 350 mg/mL.^63^ A higher internal phase volume led to higher-density materials due to the decreased pore volume. Moreover, Manzocco et al. determined that the freeze-dried whey protein isolate aerogel had 220 mg/mL density, while the sample prepared using supercritical drying had 290 mg/mL density.^64^ The porosity of emulsion-templated materials containing poly­(hydroxybutyrate-co-valerate) had a density ranging from 196 to 310 mg/mL.^58^

### 3.5 Residual Moisture Content

The residual moisture content of samples was evaluated as the percentage of water loss during the drying of samples. This parameter is essential to shelf stability. A lower moisture content reduces microbial growth risk, potentially minimizing or eliminating the need for preservatives, which is particularly advantageous for sensitive-skin products.

The resulting moisture content of the freeze-dried emulsion ranged from ∼2.32 to ∼10.89% (Figure 9). Its values significantly depended on the aqueous/oily phase ratio, namely, the more the oily phase amount, the lower is the moisture content of materials. The highest moisture content was observed for the sample containing 1% WPI and 1% glycerin at a 5/95 mixing ratio (Figure 9A). Matrices prepared with a 15/85 mixing ratio had a ∼3.64–3.90% moisture content with no significant differences. The moisture content of freeze-dried materials depends on biopolymer concentrations and, hence, the matrice network. WPI forms a heat-induced gel network influenced by the degree of protein hydration and unfolding.^65^ Therefore, the higher the WPI concentration, the lower is the moisture content.^66^ Meanwhile, sodium alginate’s ability to retain water is attributed to its carboxyl groups.^67^ Materials based on WPI and alginate were reported to have a moisture content of 6.50%.^68^

**Figure 9:**
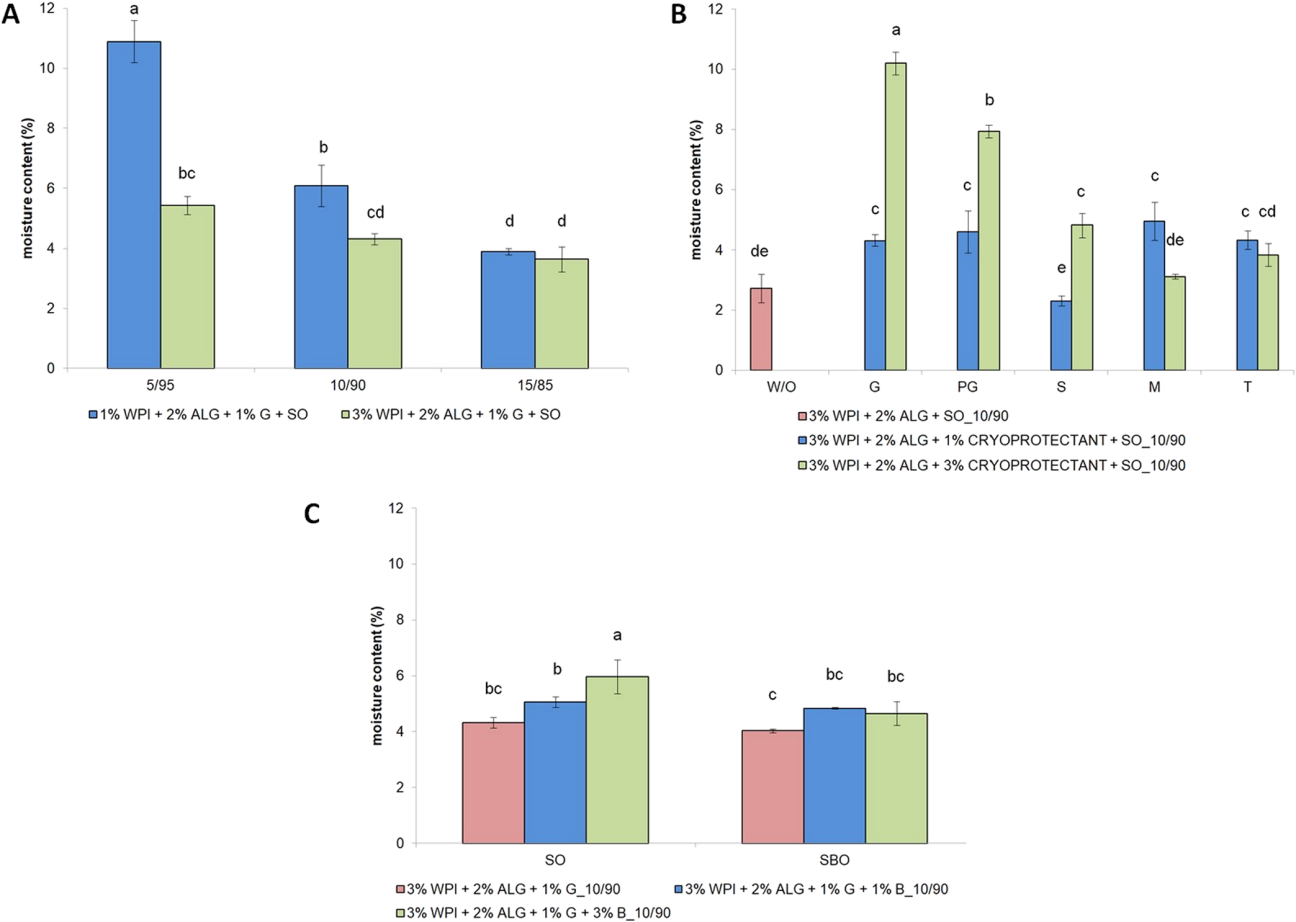
Residual moisture content of freeze-dried emulsions with altering: (A) concentration of whey protein isolate at different oily-to-aqueous phase mixing ratios; (B) type and concentration of cryoprotectants (as well as a sample without the addition of cryoprotectants); and (C) type of oil and concentration of beeswax. Bars not sharing the same letter are significantly different (*p* ≤ 0.05).

Furthermore, the residual moisture content was also significantly influenced by the concentration and type of cryoprotectants; the highest values were noted for samples with 3% glycerin (∼10.20%) and propylene glycol (∼7.94%) (Figure 9B) due to their strong hydrogen bonding capacity, which hindered complete moisture removal during sublimation, while the lowest residual moisture content of all samples was observed for materials with 1% sorbitol. A low moisture content was also detected for materials not modified with the addition of cryoprotectants (∼2.73%). Different cryoprotectants vary in hygroscopicity, influencing the amount of the residual moisture content retained after freeze-drying.^69,70^ The lower moisture content of samples containing sorbitol, mannitol, and trehalose may be attributed to their lower hygroscopicity, resembling to absorb moisture from the environment compared to glycerin and propylene glycol. Furthermore, glycerin is highly hygroscopic, which can result in the resorption of moisture. However, trehalose is considered a preferable cryoprotectant for biomolecules due to its lack of internal hydrogen bonds, allowing for more flexible hydrogen bond formation during freeze-drying.^71^

Altering the oil type and addition of beeswax contributed to the moisture content varying from ∼4 to ∼6% (Figure 9C). The composition of the oil phase, namely, the different types of oil, influences water distribution within the freeze-dried emulsion. Sea buckthorn oil is rich in polyunsaturated fatty acids, increasing oil polarity, which may facilitate better water dispersion and removal during freeze-drying.^72^ The presence of beeswax in samples may contribute to a higher residual moisture content due to its hydrophobic nature.^73,74^ Therefore, beeswax may entrap water within the freeze-dried matrix by limiting the diffusion and sublimation of water molecules. The precise selection of cryoprotectants and the optimization of biopolymer ratios, in conjunction with appropriate emulsifier and oil phase compositions, govern the extent of water immobilization and sublimation dynamics during the drying process, thereby critically impacting the resulting moisture content.

### 3.6 General Discussion

Traditional water-based emulsions dominate personal care products, although water as their base component offers minimal skincare benefits while consuming significant resources. The development of sustainable, biopolymer-based skincare products presents an innovative approach to reducing water usage and enhancing the performance of cosmetic materials. This study demonstrates the feasibility of freeze-dried emulsions formulated with biopolymers (sodium alginate and whey protein isolate), cryoprotectants (glycerin, propylene glycol, sorbitol, mannitol, and trehalose), oils (sunflower oil and sea buckthorn oil), beeswax, and emulsifiers (Span 80). Since all ingredients are either food-grade biopolymers, plant-derived oils, or cosmetic-grade waxes and polyols, commonly used in topical formulations, their established regulatory status and history of safe cosmetic use support their expected safety for cosmetic and dermatological applications.

The physicochemical properties of the freeze-dried emulsions were significantly influenced by factors such as the WPI concentration, aqueous-to-oily phase ratios, and the type and concentration of cryoprotectants, oils, and beeswax. The materials exhibited promising porosity (59–95%) and density variations (116–308 mg/mL), contributing to their lightweight nature and efficient reconstitution. Furthermore, the low residual moisture content (2.3 to 10.9%) enhances these emulsions’ stability and shelf life. The mechanical properties of the obtained materials ranged from 240 kPa to 1.7 MPa, demonstrating their robustness for application in skincare. These properties suggest that the FD emulsions maintain integrity during handling and storage, making them suitable for practical use in cosmetic formulations. One of the primary advantages of freeze-dried emulsions is their potential to reduce microbial growth. Traditional water-based emulsions require preservatives to prevent contamination; however, the removal of water from the formulation minimizes microbial growth. This makes the freeze-dried emulsions particularly beneficial for individuals with allergies or sensitivities to preservatives.

Although freeze-drying is known to be energy-intensive and may pose scalability challenges in industrial cosmetic production, several strategies can be employed to mitigate these limitations. For instance, integrating energy recovery systems in freeze-drying equipment or combining freeze-drying with predrying methods (such as microwave-assisted drying) can significantly reduce the total energy consumption. Process optimization through batch scheduling and load maximization can also improve the energy efficiency. Despite the initial energy costs, freeze-dried emulsions offer distinct advantages that may offset these inputs in a full lifecycle analysis. Key environmental benefits include reduced water consumption (water sublimed during freeze-drying can be reused in the next step of production, decreasing overall water usage), lower packaging waste (the dry form of these emulsions reduces the need for plastic packaging, as they are lighter and more compact than traditional emulsions), and efficient transport and storage (the reduced mass and volume of freeze-dried emulsions facilitate more efficient logistics, leading to a lower carbon footprint in distribution). The development of freeze-dried emulsions represents a significant advancement in cosmetic chemistry and materials science. Upon contact with a minimal amount of water just before application to the skin, the freeze-dried materials rapidly reconstitute into soft, gel-like emulsions, consistent with their original composition of biopolymers, oils, and humectants. Such textures are particularly desirable in cosmetic and dermatological applications, offering favorable spreadability, skin adherence, and a pleasant sensory profile during topical use. This novel formulation method offers several advantages over traditional emulsions, including an extended shelf life due to reduced microbial growth, increased stability under varying storage conditions, and the potential for customized skincare solutions by varying the compositions of biopolymers, cryoprotectants, oils, and active ingredients. When considering the broader environmental and economic context, these benefits position freeze-dried emulsions as a promising, sustainable alternative in the cosmetic industry.

The findings of this study underscore the potential of freeze-dried emulsions as a sustainable and functional alternative to conventional skincare products by combining existing technologies with optimized formulations. Their successful implementation could revolutionize the personal care industry, aligning with the growing consumer demand for eco-friendly and high-performance skincare solutions. However, formulation optimization, such as exploring additional biopolymers and active ingredients, rehydration behavior, long-term stability studies, and evaluating biophysical skin parameters using probands, remains a key area required for future investigation to enhance the performance of FD emulsions.

## 4 Conclusions

In conclusion, freeze-dried emulsions using sodium alginate and whey protein isolate offer a promising approach for sustainable cosmetic and dermatological products. This study explored cryoprotectants, oils, and beeswax to refine the preparation method and enhance the product quality through physicochemical characterization. Optimizing the homogenization time and speed was key to achieving a desirable droplet size distribution. Prepared freeze-dried emulsions had a complex porous structure, and their physicochemical properties significantly depended on the oily-to-aqueous phase mixing ratio, concentration of WPI, type and concentration of the cryoprotectant, type of oil, and the addition and concentration of beeswax. Different concentrations of WPI did not affect the samples’ porosity and density. At the same time, materials with lower amounts of WPI had a lower oily droplet size and span, compressive strength, and higher moisture content. The higher content of the oily phase in the emulsion composition led to a decrease in porosity and residual moisture content as well as an increase in density. Although the type and concentration of the added cryoprotectant had a slight difference for the porosity, span, and oily droplet size, a 3% addition of glycerin and propylene glycol led to higher values of density and residual moisture content, whereas a higher Young’s modulus was observed for samples with mannitol, trehalose, and 1% addition of propylene glycol and sorbitol. The addition of beeswax resulted in larger oily droplets with a narrow distribution, a higher Young’s modulus, and lower porosity and density in samples containing sea buckthorn oil and higher moisture content in materials with sunflower oil. The characterization results indicate that the physicochemical properties of these biopolymer-based freeze-dried emulsions contribute to their potential for an extended shelf life and reduced microbial growth. Additionally, these formulations offer environmental benefits by reducing water usage and plastic packaging waste, supporting the shift toward more sustainable cosmetic technologies. Overall, this research highlights the potential of freeze-dried biopolymer emulsions as tailorable, eco-friendly alternatives in the cosmetic and dermatological industries.

## Data Availability

The original contributions presented in this study are included in the article. Further inquiries can be directed to the corresponding author.
